# The Vivitrol pilot program (VPP): initial quantitative findings from an extended-release naltrexone study

**DOI:** 10.1186/1940-0640-10-S1-A54

**Published:** 2015-02-20

**Authors:** Traci Rieckmann, Katherine T Garvey, Priya Srikanth, Laura Andrich, Jessica Gregg

**Affiliations:** 1 Department of Public Health & Preventive Medicine, Oregon Health & Science University, Portland, OR, 97239-3098, USA; 2 Hooper Detoxification and Stabilization Center, Portland, OR, 97232, USA

## Background

In 2010, the FDA approved the use of extended-release injectable naltrexone (Vivitrol©) for the treatment of opioid dependence. Preliminary evidence from prior studies indicates that monthly injections of Vivitrol are effective in treating opioid dependence after initial detoxification. This longitudinal exploratory study measures demographics, medication adherence, sobriety (urinalysis), utilization of care, and mental and emotional states in patients who were treated for opioid dependence with injectable naltrexone at an addictions stabilization center.

## Materials and methods

Participants involved in the VPP completed surveys upon intake and before and after each monthly injection. Participants included patients who completed detoxification and initiated injectable naltrexone treatment (n = 71) compared to patients who dropped out of the protocol against medical advice (AMA) (n = 73). Data were analyzed using STATA (v12); Pearson’s chi-square and Fisher’s exact tests for categorical variables; and *t*-tests for continuous variables.

## Results

Quantitative results indicate that more patients who completed detoxification and received at least the first injection had previous medication-assisted treatments (MAT) program experience compared to patients who left AMA (72% vs. 58%). Participants with prior knowledge of injectable naltrexone were more likely to complete detoxification and receive an injection compared to treatment-naïve patients (61.19% vs. 45.07%). Patients were more likely to engage in group sessions with each injection (first injection 33%; second 82%) Between the first and second injections, the percentage of participants receiving emergency care decreased (35.29% vs. 27.91%).

## Conclusions

The findings from this study indicate that previous knowledge of extended-release naltrexone and prior MAT experience may influence the initiation and commitment to use of this MAT. Patients who remained in the program engaged more actively in group sessions, had higher senses of control over their drug use, and had lower rates of utilization for urgent care.

